# Should we prescribe oral metronidazole or probiotics for acute gastroenteritis in dogs?

**DOI:** 10.18849/ve.v7i2.393

**Published:** 2022-05-18

**Authors:** Emily Moore+, Wanda Gordon-Evans+

**Keywords:** STRESS

## Abstract

There is an erratum to this paper published in Veterinary Evidence Vol 7, Issue 2 (2022): 10.18849/VE.V7I2.626

PICO question

In dogs with acute gastroenteritis, is metronidazole faster, slower, or comparable in resolving clinical signs when compared to probiotic administration?

Clinical bottom line

Category of research question

Treatment

The number and type of study designs reviewed

Five studies total, all were blinded, randomised controlled trials

Strength of evidence

Moderate

Outcomes reported

The use of probiotics as a treatment for acute, uncomplicated diarrhoea in dogs may improve clinical signs faster when compared to a placebo, but showed no difference when compared directly to metronidazole. Metronidazole, when compared to a placebo, produced mixed results with one study finding that treatment with metronidazole did significantly reduce the time to resolution of diarrhoea, while another study found the difference with placebo was not significant

Conclusion

Based on the evidence evaluated, the use of oral metronidazole will not decrease time to resolution of clinical signs in cases of acute, uncomplicated diarrhoea in dogs when compared to probiotic administration and thus should not be a first-line treatment in such cases

How to apply this evidence in practice

The application of evidence into practice should take into account multiple factors, not limited to: individual clinical expertise, patient’s circumstances and owners’ values, country, location or clinic where you work, the individual case in front of you, the availability of therapies and resources.

Knowledge Summaries are a resource to help reinforce or inform decision making. They do not override the responsibility or judgement of the practitioner to do what is best for the animal in their care.

## Clinical scenario

You are presented with an 18 month old, female spayed Labrador Retriever with a 1 day history of watery, unformed stool with increased frequency. The patient does not have a previous history of diarrhoea. Physical examination is normal, aside from faecal staining around the anus; her appetite and behaviour are unchanged to mildly decreased. Faecal float and smear tests are negative for parasites and there is no apparent blood in the faecal sample.

The owner would like a treatment that will return the dog’s stools to normal consistency in the shortest amount of time and asked about prescribing metronidazole, as this has worked for her other dogs with similar presentations previously. You know that not all cases of acute gastroenteritis need to be treated with antimicrobials and will resolve with time, but the owner wants a treatment today. You wonder if prescribing a probiotic / synbiotic would be as efficacious at treating the acute diarrhoea as metronidazole is and if this would be the better option for today’s visit.

## The evidence

While the Shmalberg et al. (2019), Langlois et al. (2020), Herstad et al. (2010), Nixon et al. (2019), and Kelley et al. (2009) studies were all randomised controlled clinical trials, the evidence directly comparing the time for resolution of clinical signs in dogs treated for acute gastroenteritis is inconclusive. Shmalberg et al. (2019) is the only study that compared both treatments directly, but was underpowered and did not show statistical difference for the two treatments when compared to each other and to no intervention. The Herstad et al. (2010), Nixon et al. (2019), and Kelley et al. (2009) studies compared time to resolution of clinical signs with probiotics compared to placebo and suggest that there is faster resolution of clinical signs when a dog is treated with probiotics in cases of acute, uncomplicated diarrhoea. Langlois et al. (2020) reported that metronidazole may shorten the course of diarrhoea by 1.5 days. However, this study used an unvalidated faecal scoring system and small sample size. Based on the results of Shmalberg et al. (2019) and Langlois et al. (2020), there is no unequivocal evidence that metronidazole reduces the duration of clinical signs associated with acute, uncomplicated diarrhoea, and thus should not be used as a first-option treatment in such cases.

## Summary of the evidence

**Shmalberg et al. (2019):** 

Population:	Client-owned dogs that presented for acute diarrhoea (≤7 days) with or without vomiting, between 4–45 kg, and lacking any clinically relevant comorbidities (endocrinopathies, organ dysfunction, immune-mediated disease, suspected pancreatitis). Excluded were dogs with signs consistent with severe acute haemorrhagic diarrhoea syndrome (significant dehydration, hypovolemia, large volumes of haemtochezia).
Sample size:	63 dogs enrolled; 60 dogs completed study.
Intervention details:	There were three groups: placebo (20 dogs), probiotic treatment (20 dogs), and metronidazole treatment (20 dogs). Synbiotic (classified as probiotic in original article) treatment group: Vital VetÔ (Vital Planet, Palm Harbor, FL, USA) probiotic capsules: Probiotic composition: *Bifidobacterium bifidum* VPBB-6, *Bifidobacterium longum* VPBL-5, *Bifidobacterium animalis* VPBA-4, *Bifidobacterium infantis* VPBI-6, *Lactobacillus casei* VPLC-1, Levilactobacillus* brevis* VPLB-5, Limosilactobacillus* reuteri* VPLR-1, and *Lactobacillus bulgaricus* VPLB-7. Prebiotic composition: 425 mg organic acacia gum and fructo-oligosaccharides. 30 billion colony forming units (CFU) given by mouth twice daily for 10 days. Metronidazole treatment group: Metronidazole powder given in gelatin capsules: Oral metronidazole capsules given twice daily for 10 days. Dogs 4–10 kg received 125 mg; 12.5–25 mg/kg dose range. Dogs 10.1–20 kg received 250 mg; 12.5–25 mg/kg dose range. Dogs 20.1–45 kg received 400 mg; 8.9–20 mg/kg dose range. Typical dose range for metronidazole in cases of Giardiasis is 25 mg/kg and clostridial infections is 10–15 mg/kg when given orally. Placebo group: capsules of sucrose equal in volume to probiotic and metronidazole capsules: Oral capsules given twice daily for 10 days. Rescue treatment (treatment given to dogs that still had diarrhoea after 10 days or experienced worsening frequency of diarrhoea, worsening faecal score, increased haematochezia, or worsening straining) for any dog was tylosin, 30 mg/kg by mouth twice daily for 10 days. Three dogs were eliminated from the study, one due to significant parasite burden (metronidazole group) and two due to failure of owners to give the treatment (one in the probiotic group and one in the placebo group). A predetermined sample size of n = 20 per group was determined by power analysis prior to study. Randomisation was performed using a schedule obtained from a random sorting feature of a commercial software program (Excel for Mac).
Study design:	Double blinded, placebo controlled, randomised clinical trial.
Outcome studied:	Outcome = number of days until first formed stool, as measured by a faecal score ≤3.
Main Findings (relevant to PICO question)	Treatment group did not significantly affect the duration of diarrhoea when all dogs were included. Neither metronidazole treatment nor probiotic treatment significantly reduced the number of days until the first formed stool was appreciated in the dogs. Probiotic group: Acceptable faecal score after 3.5 ±2 days (mean value with standard deviation). Metronidazole group: Acceptable faecal score after 4.6 ±4 days (mean value with standard deviation). Placebo group: Acceptable faecal score after 4.8 ±9 days (mean value with standard deviation). P-value measured between the placebo group and the two treatment groups was p = 0.17. After dogs positive for parasite ova on faecal float were removed, there was no significant difference between this finding and the findings when parasite ova positive dogs were included in the statistics (p = 0.56): Acceptable faecal score probiotic group after 3.5 ±2 days. Acceptable faecal score metronidazole group after 4.5 ± 2 days. Acceptable faecal score placebo group after 4.8 ± 3 days. No dogs needed rescue treatment.
Limitations:	The outcome was only one formed stool, which could be a sign of resolution, but could also represent an anomaly during treatment. While complete blood count (CBC), chemistry panel, faecal floatation, and polymerase chain reaction (PCR) panels were done on most dogs, some dogs received empirical treatment (fenbendazole) or supportive care (maropitant, IV or SQ fluids) prior to study enrolment. Owners received financial compensation for participating. Dogs with a low parasite burden were included in the study (dogs with a high parasite burden were excluded). The probiotic also contained prebiotics that were not found in the placebo and could have had an effect on the microbiome. Metronidazole is typically given in the form of a tablet, capsule, or oral solution when used in a non-hospital setting, so the bioavailability of the powder form is unknown in this study.

**Langlois et al. (2020):** 

Population:	Dogs with acute, uncomplicated diarrhoea ± vomiting not due to parasitism, *Giardia *spp infection, or parvoviral enteritis. Dogs >6 months with diarrhoea <7 days duration, between 4–50 kg. All dogs were up to date on core vaccinations. The study excluded dogs that received probiotics, antimicrobials, or anti-inflammatory treatments within the preceding 30 days prior to diarrhoea onset, pregnant or nursing dogs, and dogs with moderate to severe abdominal pain, complete anorexia, or moderate to severe dehydration (>8%).
Sample size:	48 dogs recruited; 31 dogs completed study.
Intervention details:	There were two groups: treatment with metronidazole (14 dogs) and no treatment (placebo) group (17 dogs): Metronidazole group: Eight dogs received either intravenous (IV) fluids (≤1 day) or subcutaneous (SC) fluids (no dose given). Six dogs received one dose of maropitant citrate (CereniaÔ, Zoetis, Kalamazoo, MI, USA). Metronidazole target dose = 10–15 mg/kg by mouth twice daily for 7 days. Metronidazole was compounded in gel capsules by Unichem Laboratories Ltd. (Mumbai, India). Placebo group: Seven dogs received either IV (≤1 day) or SC fluids (no dose given). Six dogs received one dose of maropitant citrate (CereniaÔ, Zoetis, Kalamazoo, MI, USA). Placebo dose = capsules of microcrystalline cellulose by mouth twice daily for 7 days. Rescue treatment (metronidazole) was given if diarrhoea persisted after 7 days. Any dog that presented with vomiting could receive one dose of maropitant 1 mg/kg SC (clinician’s discretion). Hematochezia (p = 0.48), fluid therapy treatment (p = 0.48), maropitant citrate treatment (p = 0.72) were not different between the two populations of dogs. A predetermined sample size of n = 15 per group was determined by power analysis prior to study. Dogs were randomised using a computer-generated log created by the pharmacist. The dog owner and clinician were blinded to treatment. 14 dogs were excluded from the study. 10 dogs were excluded for gastrointestinal parasitism, two dogs for inability to obtain sufficient faeces for faecal testing, one dog for normal faecal score prior to therapeutic initiation, and one for ultrasonographic evidence of acute pancreatitis. Three dogs were removed from the study. One dog for severe vomiting (test population), one dog for failure to provide faecal scores and return at day 7 for diagnostics, and one for failure of faecal scoring and adherence of dietary guidelines.
Study design:	Double blinded placebo controlled randomised clinical trial.
Outcome studied:	Outcome = time in days until resolution of diarrhoea, measured as two consecutive faecal scores ≤4.
Main Findings (relevant to PICO question)	Metronidazole significantly reduced the number of days until resolution of diarrhoea (p = 0.04) in dogs when compared to the placebo group. Metronidazole population resolution of diarrhoea average (mean and standard deviation) = 2.1 ±6 days. 13/14 dogs receiving metronidazole had resolved diarrhoea by day 4. Placebo population resolution of diarrhoea average (mean and standard deviation) = 3.6 ±1 days: 2/17 dogs in the control group received metronidazole due to persistent diarrhoea at day 7.
Limitations:	Bristol scoring system is not validated in dogs nor is it commonly used, but it is validated and commonly used in human diarrhoeal studies. All dogs ate their normal diet, although one dog was excluded due to lack of owner compliance. Dogs with persistent diarrhoea at day 7 were included in statistical analysis as ‘resolved at day 7’ for statistics. Only 18/31 dogs had biochemical analysis or further diagnostic work up (abdominal US). Lead author is affiliated with Zomedica, Inc. Small sample size.

**Herstad et al. (2010):** 

Population:	Dogs with acute self-limiting gastroenteritis. The dogs had either diarrhoea or diarrhoea and vomiting. The study excluded dogs with diarrhoea ± vomiting for over 2 weeks, dogs treated with a probiotic product within a month of presentation, and dogs that required hospitalisation.
Sample size:	36 dogs; it was not stated if any dogs did not complete or were withdrawn from the study.
Intervention details:	The study had two groups: treatment with probiotics (15 dogs) and no treatment (placebo) group (21 dogs): Probiotic treatment = ZooLac Propaste Ô (Chem Vet A/S, Denmark): Probiotic composition: *Lactobacillus farciminis*, *Pediococcus acidilactici, Bacillus subtilis*, *licheniformis*, and thermo-stabilised *Lactobacillus acidophilus*. Placebo treatment = pasta-base with vegetable oil, lecithin, and E551b (stabiliser). 22 dogs also presented with vomiting: 12 in placebo group; 10 in probiotic group (no statistical analysis provided). Doses for both placebo and probiotic paste was based on weight: 1 mL for 1–10 kg; 2 mL for 10–25 kg; 3 mL for 25–50 kg. Given by the owner by mouth three times daily (every 8 hours) until normalisation of stools. First dose was a double dose in both placebo and probiotic group. Randomisation protocol was not described, only stated that there was block randomisation. Calculation of a predetermined group size (n) was not performed prior to study enrolment. No adverse effects in either group were reported.
Study design:	Double-blind placebo control randomised clinical trial.
Outcome studied:	Outcome = day (whole or half) until last abnormal stool and days until normalisation of stool.
Main Findings (relevant to PICO question)	Probiotic treatment was a significant factor for time to diarrhoea resolution (p = 0.045) but was not significant for time until resolution of signs if diarrhoea and vomiting were both present (p = 0.55). The time to first normal stool after starting treatment was not significant between the two groups (p = 0.14). Probiotic group: Time until last abnormal stool: 1.3 days (95% confidence interval (CI): 0.5–1). Time until last clinical sign (vomiting or diarrhoea): 1.4 days (95% CI: 0.5–4). Time until first normal stool: 2.9 days (95% CI: 2.1–7). Mean duration of vomiting: 0.9 days (95% CI: 0.5–3). Placebo group: Time until last abnormal stool: 2.2 days (95% CI: 1.3–1). Time until last clinical sign (vomiting or diarrhoea): 2.2 days (95% CI: 1.4–1). Time until first normal stool: 3.4 (95% CI: 2.6–2). Mean duration of vomiting: 1.2 days (95% CI: 0.2–2). Both probiotic and placebo groups had significantly reduced number of stools during the first 3 days of treatment (p ≤0.01), but were not different from each other (P = 0.19). Both probiotic and placebo groups had significantly reduced number of vomiting incidences during the first 3 days of treatment (p ≤0.01), but were not different from each other (p ≥0.16).
Limitations:	Faecal samples for bacterial and parasitic analysis were only collected from some patients, not all, and only at the initial enrolment in the clinical trial. There was no standardised way to assess normalisation of stool consistency, only done by owner opinion and relied upon owner’s recollection during interview on day 4 or 8. No clearly stated definition of ‘normalised stool’, normalisation of stool was defined by owner’s interpretation and was only subjective. Small sample size. All of the products used (probiotics and placebo paste) were supplied by the manufacturing company directly.

**Nixon et al. (2019):** 

Population:	Client owned dogs with acute diarrhoea ≥1 occurrences within 24 hours of presentation to primary care veterinarian. Dogs were excluded if their signs were deemed unsuitable for conservative management, if they received antimicrobial or probiotic treatment within 4 weeks before day 0 of enrolment. Dogs with comorbidities that did not affect diarrhoea were included in the study.
Sample size:	148 dogs initially enrolled, 118 dogs remained with no dosing errors, 107 dogs completed the entire study.
Intervention details:	The study consisted of two groups: Synbiotic (classified as probiotic in original article) group (57 dogs) and a placebo group (61 dogs). Treatment group = *Enterococcus faecium *4b1707, Preplex® (PKA, Protexin Veterinary, Somerset, UK) prebiotics, combined kaolin and montmorillonite clay, psyllium, pectin, and beta glucan: Given by mouth three times daily (every 8 hours). Placebo group = placebo composed of soya oil, colloidal silica, dextrose, liver flavour: Given by mouth three times daily (every 8 hours). Dosing by body weight for probiotic and placebo group: <5 kg = 2 mL 5–15 kg = 2 mL 15–30 kg = 5 mL 30–45 kg = 7 mL ≥45 kg = 10 mL All dogs ate Hill’s® i/d (Topeka, Kansas, USA) for duration of study. A predetermined group size of 43 cases based on a power calculation. Randomisation was performed using block randomisation with a block size of 2 stratified by site. 41 dogs did not complete the study. 10 were included inappropriately (no episode of diarrhoea), 10 had dosing errors (inappropriate therapy dose or failure to accept new diet), 10 did not receive a confirmed dose of therapy, and 11 for worsening or non-improvement of signs. The number of dogs excluded from each group was not specified.
Study design:	Double-blinded, placebo controlled, randomised clinical trial.
Outcome studied:	Outcome definition: time until passage of three consecutive faeces of normal consistency (faecal score ≤3) measured from initial presentation to the veterinary clinic within 10 days of initial study enrolment.
Main Findings (relevant to PICO question)	Probiotic treatment was significant (p = 0.008) in decreasing time to resolution of diarrhoea as measured as the passage of three consecutive normal stools (faecal score ≤3). Probiotic group duration of diarrhoea median time: 32 hours (range 2–118 hours; n = 51). Placebo group duration of diarrhoea median time: 47 hours (range 4–167 hours; n = 58). Fewer dogs in the probiotic group required additional medical intervention when compared to the placebo group. Additional treatment could include antiemetics, gastrointestinal (GI) motility modulating drugs, antimicrobials, and corticosteroids. Probiotic group requiring additional treatment: two dogs with five total treatments. Placebo group requiring additional treatment: nine dogs with 15 total treatments. The dogs were withdrawn from the study and there was no significant difference in time until withdrawal between the probiotic group (61 hours ± 41 hours) and the placebo group (45 hours ± 3 hours) and there was not a significantly different relative risk of needing additional medical treatment in either group.
Limitations:	While the data is significantly different, the result may not be clinically different (only 0.5 day) for the owner. No diagnostic testing to rule out infectious causes of diarrhoea or unknown comorbidities, which could impact the responsiveness of the diarrhoea to a probiotic alone. While the Mann-Whitney test was used to determine differences in median age between the group was not significantly different, the value was very close to P and may confer a clinical difference. The median age of the treatment group was 45 months, while the median age of the placebo group was 24 months. Dogs were allowed to be removed from the trial if they needed additional medical intervention; more were removed from the placebo group and could represent attrition bias. Dogs that missed one dose in a day in either the placebo or treatment group were not excluded, which may impact results given the timing of resolution of diarrhoea was measured in hours and was around four doses for the treatment group (specific numbers were not given). This lack of strict compliance may mimic the realities of daily life (in respect to client compliance) therefore, it is not unreasonable to still include these dogs. The placebo had a different composition than the probiotic; it would be a stronger study if the placebo was the same composition as the probiotic without the probiotics and prebiotics. Two authors were employees of the company that produced the probiotic.

**Kelley et al. (2009):** 

Population:	Young, adult dogs that presented to their veterinarian with acute idiopathic diarrhoea with a stool score of 4; all dogs from a guide dog organisation from a single training campus. Excluded dogs that had other medical conditions, were being treated with other medications, or had stool scores <3.
Sample size:	45 recruited; 36 met inclusion criteria, 31 dogs completed study.
Intervention details:	The study consisted of two groups: Probiotic group (13 dogs) and a placebo group (18 dogs): Probiotic group = canine derived *Bifidobacteerium animalis* AHC7 (Procter and Gamble Pet Care, Lewisburg, OH, USA) in cocoa butter treats: Dose = 2 x 10^10^ colony forming units (CFU) / day by mouth twice daily for 14 days or until resolution of diarrhoea. Placebo = same vehicle as the test agent without probiotic (cocoa butter treats): Placebo dose = by mouth twice daily for 14 days or until resolution of diarrhoea. Both groups ate either EukanubaÔ (Procter & Gamble, Cincinnati, OH, USA) or IamsÔ (Procter & Gamble, Cincinnati, OH, USA) maintenance diets. At the beginning of the study, any dog could be prescribed metronidazole at DVM’s discretion for number of abnormal stools, degree of stool looseness, health of dog, and if dog was housed nearby outbreaks of diarrhoea. Metronidazole dose = 750 mg, by mouth twice daily for 7 days. Nine dogs in the control group received metronidazole. Four dogs in the treatment group received metronidazole for GI disease. Five dogs were excluded from statistical analysis – Four dogs had missing stool scores; one dog had been previously enrolled in the study. The kennel staff performing the stool scoring were blinded to the dog’s treatment group. Randomisation protocol was not stated.
Study design:	Blinded, placebo controlled randomised clinical trial.
Outcome studied:	Outcome = time, in days, until resolution of diarrhoea. Resolution of diarrhoea was defined as stool scores that improved from 4 to ≤2 and remained ≤2 for at least 5 consecutive days.
Main Findings (relevant to PICO question)	Treatment with probiotic significantly reduced days to resolution of diarrhoea when compared to the placebo group in all dogs, and in both German Shepherds and Labrador Retrievers, while the Labrador-Golden Retrievers or Golden Retrievers did not show a significant difference due to small sample size. Probiotic group all dogs: mean days to resolution of diarrhoea was 3.9 ±3., Placebo group all dogs: mean days of resolution of diarrhoea was 6.6 ±7 days. P-value for all dogs, p = <0.01. Probiotic group German Shepherds: mean days to resolution of diarrhoea was 3.3 ±9. Placebo group German Shepherds: mean days of resolution of diarrhoea was 7.7 ±8 days. P-value for German Shepherds, p = 0.03. Probiotic group Labrador Retrievers: mean days to resolution of diarrhoea was 3.3 ±3. Placebo group Labrador Retrievers: mean days of resolution of diarrhoea was 6.3 ± 2.4 days. P-value for Labrador Retrievers, p = 0.05. Probiotic group Labrador-Golden Retrievers: mean days to resolution of diarrhoea was 5.3 ±5. Placebo group Labrador-Golden Retrievers: mean days to resolution of diarrhoea was 6.0 ±5 days. P-value for Labrador-Golden Retrievers, p = 0.73. Probiotic group Golden Retrievers: mean days to resolution of diarrhoea was 1.0 ±0. Placebo group Golden Retrievers: mean days of resolution of diarrhoea was 6.0 ±0 days. P-value for Golden Retrievers not calculated, only one dog per group. 5/13 dogs in the treatment group received metronidazole: Mean days of resolution of diarrhoea in probiotic dogs that did not receive metronidazole: 3.33 ±26 days (based on reviewer’s calculation). 9/18 dogs in the control group received metronidazole: Mean days to resolution of diarrhoea in placebo dogs that did not receive metronidazole: 6.11 ±88 days (based on reviewer’s calculation). When dogs receiving metronidazole were excluded from the study, resolution of diarrhoea by day 4 occurred in 3/9 dogs in the control group and 7/9 dogs in the probiotic group – statistical analysis was not presented.
Limitations:	Funded by Procter and Gamble Pet Care, which produced the probiotic used in the treatment group. All dogs were from the same facility, which could be a confounder (a bias in management or population). Dogs receiving metronidazole were included in the statistics and were more commonly in the control group. Giardia positive dogs were included in the statistics and received metronidazole (standard of care for giardiasis); these dogs should have been eliminated from the study as the cause is infectious and not idiopathic. Small samples sizes when considering the data with and without the metronidazole treated dogs. Very small sample size in some subgroups.

## Appraisal, application and reflection

Dogs with cases of acute gastroenteritis are commonly seen at first opinion clinics (Singleton et al., 2019). Two commonly used options for treatment of dogs with acute, uncomplicated diarrhoea are systemic antimicrobials, mainly metronidazole (although in the UK potentiated amoxicillin is more commonly used), and nutraceuticals, including various formulations of probiotics / synbiotics (Singleton et al., 2019). Many cases of acute diarrhoea are self-limiting (Shmalberg et al., 2019), yet many mild cases are still treated with metronidazole (Singleton et al., 2019). The continued prescribing of metronidazole may be related to a perception that metronidazole administration resolves a patient’s diarrhoea in a more timely manner and satisfies an owner’s expectation of medication administration. However, there is also a rising concern about veterinary use of antimicrobials and the effects on human health (Herstad et al., 2010; and Prescott, 2019).

The use of antimicrobials in all fields of veterinary medicine can have effects on the microbial population, which in turn can impact human health. Research in companion animals shows the close relationship between humans and their pets allows for the transfer of microbes between the species, thus increasing the potential for transfer of methicillin-resistant bacteria between the human and pet. With increased antimicrobial resistance, the treatment of infections may become more challenging in both human and veterinary medical fields (Lloyd & Page, 2018). Thus an alternative treatment regimen needs to be established for uncomplicated acute diarrhoea in the veterinary population that both alleviates the patient’s condition and placates the client. The practice of prescribing probiotics for treatment of acute diarrhoea in veterinary patients may offer an alternative solution.

The direct study of the time to resolution of clinical signs (diarrhoea) using metronidazole compared to probiotics has not been studied adequately in dogs that present for acute, uncomplicated diarrhoea. Only one study directly compares time to resolution of diarrhoea in dogs treated with metronidazole or probiotics, while the remaining data must be extrapolated from studies comparing either oral metronidazole use to a placebo, or probiotic use to a placebo.

Shmalberg et al.’s (2019) study directly comparing treatment with metronidazole, a probiotic product, and a placebo, found there was no significant reduction in time to resolution of acute diarrhoea when the dog received a probiotic or metronidazole. The metronidazole-treated group had a very similar average to the placebo-treated group, but probiotic average days until resolution of diarrhoea was 1 day shorter than both, which may be clinically important for a client.

Langlois et al. (2020) found that metronidazole treatment for cases of acute, uncomplicated diarrhoea did have a significant impact on time to resolution of diarrhoea when compared to a placebo. Metronidazole treatment was found to resolve clinical diarrhoea 1.5 days sooner than the placebo. A limitation of the study is that the participants’ diet was not controlled, which is also a mainstay of common treatment (Singleton et al., 2019). The sample sizes were small for this study as well.

Herstad et al. (2010), Nixon et al. (2019), and Kelley et al. (2009) performed randomised clinical trials comparing the use of probiotics and a placebo to evaluate timing of resolution of clinical signs. While Herstad et al. (2010) found no significant improvement in the probiotic treated group over the placebo group when the dogs presented with both vomiting and diarrhoea, Herstad et al. (2010), Nixon et al. (2019), and Kelly et al. (2009) found that probiotic administration did provide an improvement over the placebo by reducing time in days to resolution of diarrhoea. Herstad et al. (2010) did not provide clients with a standard faecal scoring system to use and only based faecal consistency on client opinion, while also not eliminating parasitic or infectious causes of diarrhoea. The placebo used in the Nixon et al. (2019) study varied in composition from the probiotic formulation, which could be a confounding factor. Again, Nixon et al. (2019) failed to remove dogs with infectious causes of diarrhoea as well. The population of dogs used in the Kelley et al. (2009) study may not be representative of the general population of dogs but does remove confounding differences in environment and owner compliance as seen in Shmalberg et al. (2019), Langlois et al. (2020), Herstad et al. (2010), and Nixon et al. (2019). Kelley et al. (2009) found that probiotic administration reduced time of resolution of diarrhoea by 2.5 days, which may be significant to many owners.

Overall, the results of studies by Shmalberg et al. (2019), Langlois et al. (2020), Herstad et al. (2010), Nixon et al. (2019), and Kelley et al. (2009) provided weak evidence for the efficacy of metronidazole compared to placebo for treatment of acute, uncomplicated diarrhoea. Given the lack of clear benefit for resolution of diarrhoea with a course of metronidazole, the long-term effects of antimicrobials (specifically metronidazole) on the microbiome of the gastrointestinal tract must also be considered when prescribing antimicrobial / antibacterials as they could be unfavorable for the patient. Pilla et al. (2020) and Igarashi et al. (2014) found that oral metronidazole treatment for cases of acute diarrhoea led to changes in the gastrointestinal microbiome composition, particularly bacteria responsible for bile acid metabolism, that persisted for up to 1 month post antibacterial treatment in some dogs. During oral metronidazole treatment, the faecal dysbiosis index was increased, but returned to normal after 2 weeks of metronidazole treatment (Pilla et al., 2020; and Nogueira et al., 2019). In contrast, probiotic use generally appears to be safe in veterinary species. The potential risk of probiotic use must be extrapolated from human research. The greatest apparent risk is the potential of a bacterium to become pathogenic, particularly if the intestinal barrier is not fully developed, but appeared safe when used in patients with a history of immunosuppression (either iatrogenic or infectious in origins) (Butel, 2014). Additionally, natural bacterial behaviours, such as genetic material transfer, can occur and could be detrimental if the probiotic bacterium conveyed antimicrobial resistant genetic material to the normal flora, though evidence of this occurring is lacking (Butel, 2014).

Further research needs to be performed in order to clinically answer this PICO question. Shmalberg et al. (2019) is the only study that directly compares the use of metronidazole and a probiotic product and did not show a significant advantage of either treatment compared to each other or a placebo. There is insufficient evidence to answer whether or not metronidazole affects the duration (shorter, or longer, or no difference) of clinical signs when compared to probiotic administration in cases of acute, uncomplicated diarrhoea.

Additionally, when evaluating the use of probiotics / synbiotic products as treatment to reduce the duration of acute, uncomplicated diarrhoea, the evidence remains weak. Shmalberg et al. (2019) showed that probiotics were equivalent to metronidazole in timing of resolution of diarrhoea. While Herstad et al. (2010) and Nixon et al. (2019) showed earlier time to clinical resolution of diarrhoea, they did not show a significant advantage when compared to placebo. Only Kelly et al. (2009) showed a significant reduction in days to resolution of diarrhoea, but only in certain dog breeds. Given the clinical scenario of an otherwise healthy dog presenting for acute and uncomplicated diarrhoea, prescribing probiotic / symbiotic products to satiate owner expectations (in insistence) would be a reasonable choice as the risk associated with these products is low and no long-term effects have been reported or studied.

## Methodology Section

**Search Strategy:** 

Databases searched and dates covered:	PubMed on NCBI Platform, 1999–2021.CAB Abstracts on OVID Platform, 1999–2021.
Search strategy:	PubMed:Acute AND (diarrhea OR diarrhoea OR gastroenteritis) AND (canine OR dog OR dogs) AND (metronidazole OR probiotic OR probiotics OR nutraceutical) CAB Abstracts:(Acute and (diarrhea or diarrhoea or gastroenteritis) and (dog or dogs or canine or canines) and (metronidazole or probiotic or probiotics or nutraceutical))
Dates searches performed:	19 Dec 2021

**Exclusion / Inclusion Criteria:** 

Exclusion:	Articles not in English, review articles, articles that did not measure the desired outcome, articles that studied diarrhoea that was not acute, or articles that studied diarrhoea of a known cause.
Inclusion:	Articles available in English which were relevant to the PICO. Articles had to involve more than one dog and compare the PICO pharmacologics to a placebo or each other.

**Search Outcome:** 

Database	Number of results	Excluded – Articles not in English	Excluded – Clinical review article	Excluded – Desired outcome not measured	Excluded – Studied non-acute diarrhoea	Excluded – Known cause of diarrhoea	Unable to access article	Total relevant papers
PubMed	28	1	6	6	5	5	0	5
CAB Abstracts	37	7	9	8	2	5	1	5
Total relevant papers when duplicates removed								5

## Conflict of interest

The authors declare no conflicts of interest.

## Disclaimer

Knowledge Summaries are a peer-reviewed article type which aims to answer a clinical question based on the best available current evidence. It does not override the responsibility of the practitioner. Informed decisions should be made by considering such factors as individual clinical expertise and judgement along with patient’s circumstances and owners’ values. Knowledge Summaries are a resource to help inform and any opinions expressed within the Knowledge Summaries are the author's own and do not necessarily reflect the view of the RCVS Knowledge. Authors are responsible for the accuracy of the content. While the Editor and Publisher believe that all content herein are in accord with current recommendations and practice at the time of publication, they accept no legal responsibility for any errors or omissions, and make no warranty, express or implied, with respect to material contained within. For further information please refer to our Terms of Use.
